# The Fidelity Paradox in Spinal Cord Injury: Reframing Biomechanical Mimicry and Neurobiological Relevance for Clinical Translation

**DOI:** 10.1002/cns.70929

**Published:** 2026-05-16

**Authors:** Liang Cao, Wenjun Pi, Yanjun Zhang, V. Wee Yong, Mengzhou Xue

**Affiliations:** ^1^ Department of Cerebrovascular Diseases The Second Affiliated Hospital of Zhengzhou University Zhengzhou Henan China; ^2^ Henan International Joint Laboratory of Intracerebral Hemorrhage and Brain Injury Zhengzhou Henan China; ^3^ Department of Traumatic Orthopedics The Affiliated Hospital of Guizhou Medical University Guiyang Guizhou China; ^4^ Hotchkiss Brain Institute and Department of Clinical Neurosciences University of Calgary Calgary Alberta Canada

**Keywords:** biological fidelity, immunodynamics, mechanistic fidelity matching, preclinical modeling, translational failure

## Abstract

**Background:**

Despite abundant neuroprotective successes in rodents, effective clinical therapies for spinal cord injury (SCI) remain elusive. This translational disconnect stems from a fidelity paradox. The over‐reliance on rodent models that achieve biomechanical mimicry of human trauma but lack fundamental biological equivalence.

**Methods:**

We evaluated how physical simulation masks critical interspecies divergences. Following PRISMA guidelines, we systematically searched PubMed and Web of Science (2000–2024) to assess preclinical model utilization frequencies and synthesized evidence across distinct pathophysiological pillars to evaluate cross‐species biological mismatches.

**Results:**

Our analysis reveals profound spatiotemporal dissonances across three pillars: (i) immunodynamics, where the delayed inflammatory resolution in mice misaligns with human chronobiology; (ii) lesion architecture, distinguishing rodent cystic cavitation from the fibrotic scarring characteristic of human pathology; and (iii) neural circuitry, contrasting indirect rodent corticospinal projections with the direct cortico‐motoneuronal connections essential for human dexterity. To address this, we propose a mechanistic fidelity matching framework. This paradigm shifts from indiscriminate single‐model validation to a problem‐driven matrix, selecting models based on their specific biological fidelity to targeted mechanisms—utilizing mice for scar modulation, rats for cystic repair, and nonhuman primates for fine sensorimotor recovery.

**Conclusion:**

Restoring translational credibility requires replacing the pursuit of a universal model with a hierarchical, cross‐species validation pipeline anchored in objective, mechanism‐coupled readouts.

## Graphical abstract

The manuscript attributes translational failure in spinal cord injury to a fidelity paradox: prioritizing biomechanical mimicry over biological equivalence. To bridge this gap, we introduce the mechanistic fidelity matching (MFM) framework. MFM shifts the paradigm from indiscriminate single‐model reliance to selecting species based on their specific biological alignment with the therapeutic target. This hierarchical, cross‐species pipeline restores clinical predictive power.

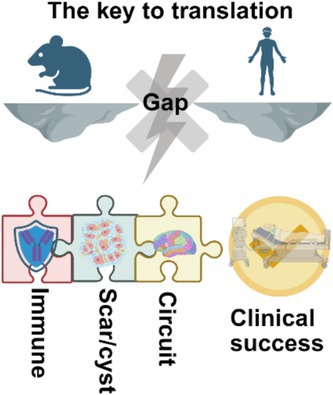

**Abbreviations**
BBB: Basso‐Beattie‐Bresnahan
CST: corticospinal tract
MFM: mechanistic fidelity matching
NHPs: nonhuman primates
PRISMA: Preferred Reporting Items for Systematic Reviews and Meta‐analyses
SCI: spinal cord injury

## 1 Introduction

Spinal cord injury (SCI) is one of the most devastating conditions in neuroscience, inflicting permanent disability upon millions of patients worldwide and imposing a heavy socioeconomic burden [1, 2, 3]. Over the past few decades, basic research has unraveled the complex pathophysiological cascades following SCI at an unprecedented rate. This has led to numerous neuroprotective and axon‐regenerative strategies that have demonstrated remarkable efficacy in animal models, continuously fueling hope for clinical translation [4, 5, 6, 7].

However, the translational dilemma from bench to bedside has become a disquieting norm in the SCI field. Countless therapies that showed potent restorative potential in animal models have repeatedly failed in human clinical trials [8, 9, 10]. While multifactorial issues—including suboptimal clinical trial design or patient heterogeneity—undoubtedly contribute to individual translational setbacks, the widespread and systemic nature of these failures points more fundamentally toward a critical flaw in the validity of our preclinical research paradigm. It compels us to re‐evaluate the cornerstone of our research: the fidelity with which animal models of SCI recapitulate human pathology.

A growing body of evidence indicates that the root of the problem lies in the biological chasm between species [11, 12]. Study show that the glial scar, which forms rapidly in rodents post‐injury, differs significantly in structure and function from the chronic scar in humans [13]. Concurrently, the response patterns of the immune system, particularly, the activation states of microglia and macrophages, do not fully align with those observed in human patients [14]. These disparities ultimately result in preclinical models that, while morphologically simulating the trauma, diverge from the human clinical condition at the level of key pathophysiological mechanisms.

Therefore, this review will first deconstruct the existing toolbox of SCI animal models, delineating their respective strengths and limitations in physically simulating human injury. We will then elucidate the critical, and often profound, biological differences between current models and human pathology from three core perspectives: the temporal mismatch in inflammatory dynamics, structural differences in the reparative microenvironment, and innate disparities in neural circuit anatomy. Finally, to move beyond the current impasse, we introduce the mechanistic fidelity matching (MFM) framework. This paradigm shifts the focus from seeking a single perfect species to selecting models based on their biological alignment with specific therapeutic targets—scarring, regeneration, or circuitry—thereby restoring predictive validity to preclinical trials.

## 2 The Current Paradigm: A Flawed Toolbox Prioritizing Physical Form Over Biological Fidelity

**FIGURE 1:**
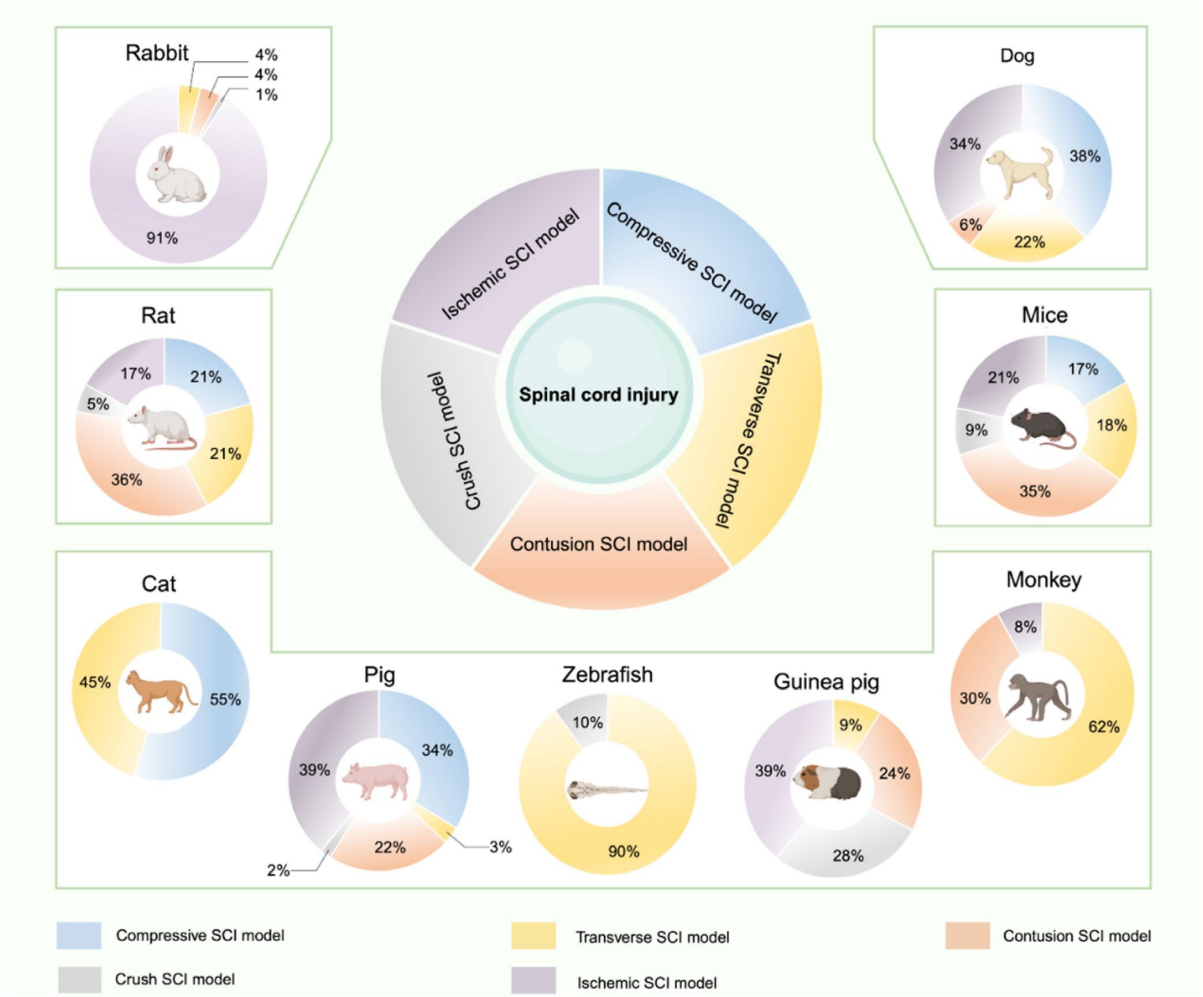
Quantitative analysis of preclinical model utilization: a data‐driven imbalance. The pie charts illustrate the distribution of common SCI models utilized across nine key species, based on a comprehensive literature search from 2000 to 2024. Percentages are accompanied by the absolute number of studies (*N*) for rigorous interpretation. Specifically, in rabbits (total *N* = 193), the most common SCI model is ischemic injury, accounting for 91% (*n* = 175), while transverse injury accounts for 4% (*n* = 8), contusion injury for 4% (*n* = 8), and crush injury for 1% (*n* = 2). In mice (total *N* = 1263), the most common SCI model is contusion injury, accounting for 35% (*n* = 442), followed by compressive injury at 17% (*n* = 215), transverse injury at 18% (*n* = 227), crush injury at 9% (*n* = 114), and ischemic injury at 21% (*n* = 265). In rats (total *N* = 3784), the most common SCI model is contusion injury at 36% (*n* = 1362), with transverse injury at 21% (*n* = 795), compressive injury at 21% (*n* = 795), crush injury at 5% (*n* = 189), and ischemic injury at 17% (*n* = 643). For guinea pigs (total *N* = 76), the most common SCI model is ischemic injury at 39% (*n* = 30), followed by transverse injury at 9% (*n* = 7), contusion injury at 24% (*n* = 18), and crush injury at 28% (*n* = 21). In cats (total *N* = 20), the most common SCI model is compressive injury at 55% (*n* = 11), with transverse injury at 45% (*n* = 9). For dogs (total *N* = 467), the most common SCI model is compressive injury at 38% (*n* = 177), followed by transverse injury at 22% (*n* = 103), contusion injury at 6% (*n* = 28), and ischemic injury at 34% (*n* = 159). In pigs (total *N* = 152), the most common SCI model is ischemic injury at 39% (*n* = 59), with compressive injury at 34% (*n* = 52), transverse injury at 3% (*n* = 5), contusion injury at 22% (*n* = 33), and crush injury at 2% (*n* = 3). For primates (total *N* = 50), the most common SCI model is transverse injury at 62% (*n* = 31), with contusion injury at 30% (*n* = 15) and ischemic injury at 8% (*n* = 4). Finally, in zebrafish (total *N* = 50), the most common SCI model is transverse injury at 90% (*n* = 45), followed by crush injury at 10% (*n* = 5).

To effectively investigate the complex pathophysiology of SCI (Figure S1) and evaluate potential therapies at the preclinical stage, a diverse array of animal models has been established [15, 16, 17]. Here, we summarize the common SCI induction methods used in nine key species: mice, rats, guinea pigs, rabbits, cats, dogs, pigs, nonhuman primates (NHPs), and zebrafish (Figure S2). Specifically, data regarding model utilization frequencies were derived from a systematic search of PubMed and Web of Science databases covering the period from 2000 to 2024, conducted in accordance with PRISMA guidelines. Search queries combined “spinal cord injury” with keywords including “contusion,” “compression,” “transection,” “ischemia,” and “crush.” From an initial yield of 8694 records, 1096 were excluded based on title/abstract screening (exclusion criteria included: non‐English literature, in vitro studies, reviews, and clinical trials). Following full‐text review, 6055 primary animal studies met our inclusion criteria, which required explicit reporting of animal species, trauma type, spinal segment, and injury stage. To minimize redundancy, multiple publications by the same research group utilizing the identical model setup were counted as a single entry. Statistical results indicate that mice and rats are most frequently subjected to contusion models; guinea pigs are often used for compression models; rabbits for ischemic models; cats and dogs primarily for compression models; pigs commonly for ischemic models; and NHPs and zebrafish predominantly for transection models (Figure 1). The core objective of these models is to replicate the injury mechanisms of specific clinical scenarios at a physical and macropathological level through controlled experimental means—that is, to pursue a simulation of the physical form of human injury. To elucidate the specific applications, advantages, and inherent limitations of each tool, we have functionally reorganized these models according to the key clinical scenarios they simulate (Figure S3).

### 2.1 Simulating Acute Closed Trauma: Replicating Instantaneous Violent Injury

Acute closed trauma, such as that resulting from traffic accidents or falls from height, is the most common clinical type of SCI. Its pathological core is a complex cascade of secondary injuries triggered by an instantaneous mechanical force [18, 19, 20]. The contusion model, which applies a rapid and transient impact to the spinal cord via a weight‐drop or a precisely controlled impactor device, is the primary tool for simulating this type of trauma [21]. It excels at recapitulating the complex pathological processes following acute injury and holds high clinical relevance, making it widely used for studying mechanisms in the acute and subacute phases and helping researchers understand post‐injury responses and recovery [21, 22, 23, 24, 25]. By adjusting factors such as impact weight, height, and velocity, researchers can precisely control the severity of the injury. This enables the rapid evaluation of the efficacy of new treatments and interventions and aids in the identification of effective drugs or therapeutic strategies [26, 27, 28, 29, 30]. The model accurately reflects the SCI mechanisms encountered in clinical settings, thereby facilitating the translation of research findings into clinical applications [31, 32]. However, a limitation is that while it allows for the swift assessment of short‐term outcomes, the long‐term effects on functional recovery may require further investigation using other models [33]. For these reasons, the contusion model is predominant in rodent studies, representing the most frequently used model in both mice (35%) and rats (36%) (Figure 1). This heavy reliance on rodents, however, masks the central flaw of the current paradigm. The model's simulation of physical form creates a translational trap by ignoring the profound biological mismatches that determine outcomes. First, the inflammatory dynamics diverge starkly: in rats, neutrophil infiltration peaks at 6 h post‐injury, whereas in mice, it is delayed and sustained, peaking between 3 and 14 days [34, 35]. An immunomodulatory drug targeting this acute 6‐h window, validated in rats, may fail to capture the optimal therapeutic window in mice, severely complicating its extrapolation to humans. Second, the reparative microenvironment is fundamentally different: rats tend to form cystic cavities, which are pathologically analogous to chronic human SCI, while mice develop a dense, fibrotic scar [13, 36]. A regenerative strategy (e.g., a biomaterial scaffold) designed to fill a cavity (rat) would be ineffective against a dense physical barrier (mouse). Thus, the field's two most common models yield contradictory data on the two most critical therapeutic targets: inflammation and scarring.

### 2.2 Simulating Chronic Compressive Injury: A Unique Window Into Slow Pathological Progression

Chronic compression, arising from conditions such as intervertebral disc herniation, epidural tumors, or spinal stenosis, involves a relatively slow injury process with progressive pathological changes. This provides a unique window for studying chronic‐phase pathology and endogenous repair mechanisms [37, 38]. The compression model, which induces injury by applying sustained pressure to the spinal cord with a specialized device, is particularly effective for simulating clinically common closed injuries like those caused by disc herniation or tumor encroachment [39, 40, 41, 42, 43]. By adjusting the applied pressure and duration, researchers can systematically control the injury severity, which facilitates the evaluation of different therapeutic approaches [44, 45]. A potential limitation, however, is its inability to fully recapitulate the complexities of other injury types, such as transection or ischemic injuries. The compression model is especially common in studies involving larger animals, such as cats (55%) and dogs (38%) (Figure 1). Additionally, the crush model induces injury by applying a brief but sustained compressive force to the spinal cord using calibrated forceps [46, 47, 48, 49, 50]. This model effectively simulates clinical scenarios of spinal cord compression caused by tumors, hematomas, or other pathological states that exert persistent pressure [51, 52]. In chronic‐phase studies, the crush model is valuable for analyzing responses related to neuro‐regeneration and repair [53, 54, 55]. It is well‐suited for long‐term observation, allowing for the assessment of various interventions and functional recovery, thereby providing deep insights into the pathological processes of chronic injury [56, 57]. A key limitation, however, is that while it effectively mimics compression, it may not fully represent the complex injury mechanisms initiated by other insults, such as transection [58]. While these models are valuable for studying chronic pathology, their translational value for functional recovery is often overestimated. The assessment of recovery in quadrupedal animals (rodents, cats, dogs) relies heavily on gross locomotor scoring (e.g., BBB score). This approach overlooks the fundamental neuroanatomical differences in the corticospinal tract (CST) [59]. These species largely lack the direct, monosynaptic CST‐to‐motor‐neuron connections that are the anatomical basis for fine, independent motor control in humans [60]. Therefore, recovery observed in these models may rely on compensatory mechanisms or reorganization of circuits that are simply not present in primates, severely limiting their predictive power for human functional restoration.

### 2.3 Simulating Penetrating and Complete Injuries: Establishing a Platform for Regeneration Research

Although clinically less common, penetrating injuries or complete spinal cord transection resulting from stab wounds or severe fractures hold an indispensable role in basic research due to their unique injury characteristics [61, 62, 63, 64]. The transection model involves the partial (hemi‐section) or complete severing of the spinal cord using microsurgical instruments [65, 66, 67, 68]. Characterized by a complete disruption of cord continuity, this model simulates severe clinical SCI scenarios, including catastrophic trauma or surgical mishaps [69, 70, 71, 72]. The primary objective of the transection model is to investigate the pathophysiological changes and repair mechanisms following the complete severance of the spinal cord. Given its definitive and severe nature, the transection model is ideal for studying axonal regeneration. It allows researchers to conduct long‐term observations postinjury, assess the efficacy of different therapeutic strategies, and evaluate the recovery of spinal cord function, making it highly suitable for chronic‐phase studies [62, 73, 74, 75]. Its main limitation, however, is that the total loss of function resulting from complete transection differs significantly from that of partial or compressive injuries. Consequently, the model may not effectively reflect the injury and recovery processes associated with partial or less severe trauma [63]. For this reason, the model is extensively used in species where the entire regenerative process can be clearly observed, such as NHPs (62%) and zebrafish (90%), the latter of which possesses a robust regenerative capacity (Figure 1). The high prevalence of this model in NHPs (62%) is a direct acknowledgment of these innate differences in neural circuit anatomy. Unlike rodents, primates possess an extensive, direct monosynaptic CST that innervates ventral horn motor neurons, a feature essential for fine motor control (e.g., independent hand movements) [60]. Therefore, for any therapy aiming to restore clinically relevant fine motor function, validation in an NHP model is not optional‐it is the only model that possesses the requisite anatomical fidelity to test the hypothesis. Studies in rodents, while useful for mechanistic discovery of regeneration, cannot predict this specific functional outcome.

### 2.4 Simulating Vasculogenic Injury: Focusing on Ischemia‐Induced Secondary Damage

In addition to direct mechanical trauma, the interruption of spinal cord blood supply resulting from aortic surgery complications, vascular malformations, or thrombosis constitutes another distinct etiology of SCI [76, 77, 78]. The ischemic model induces injury by blocking the blood supply to the spinal cord [79, 80, 81]. This is typically achieved by transiently clamping the thoracoabdominal aorta or key arteries supplying the spinal cord, thereby causing ischemia–reperfusion injury to a specific region [82, 83, 84]. The ischemic SCI model simulates clinical scenarios of interrupted spinal cord blood flow, such as that caused by vascular occlusion, aneurysm rupture, or surgical interference [85, 86, 87]. The primary purpose of the ischemic model is to investigate the pathophysiological changes and repair mechanisms within the spinal cord under ischemic conditions [88, 89, 90, 91]. The model is particularly valuable for studying the secondary neuronal injury mechanisms triggered by ischemia, such as oxidative stress, inflammatory responses, and cell death. This makes it a unique and indispensable tool for developing vasculo‐protective and anti‐ischemic drugs [88, 92]. However, like their mechanical trauma counterparts, these models are subject to the same translational chasm in inflammatory dynamics (Weapon A). The cellular and temporal profile of immune infiltration (e.g., neutrophils, macrophages) and microglial activation following ischemia–reperfusion is highly species‐specific [93, 94]. An immunomodulatory drug validated in a rabbit model, which dominates this category (91%), may target a therapeutic window or cellular cascade that does not align with that in human pathology, again highlighting the limitations of relying on a single‐species, single‐model validation approach (Figure 1). However, while the ischemic model faithfully recapitulates the hemodynamics of vascular occlusion, the species‐specific divergence in inflammatory kinetics imposes an intrinsic biological limitation on the precise definition of the therapeutic window—the decisive factor for the clinical efficacy of neuroprotective interventions. In summary, while the aforementioned models (contusion, compression, transection, and ischemia) are indispensable for mirroring the distinct biomechanical and physical triggers of SCI, they share a profound anatomical caveat. The evaluation of fine motor recovery in quadrupedal models is universally confounded by the absence of direct, monosynaptic corticospinal projections—the primary anatomical substrate for human dexterity. This inherent validity ceiling, along with other critical interspecies divergences, dictates that simulating physical form is insufficient for clinical translation. We will systematically dissect these biological chasms in the following section.

## 3 Interspecies Pathophysiological Differences as the Root of Translational Failure

**FIGURE 2:**
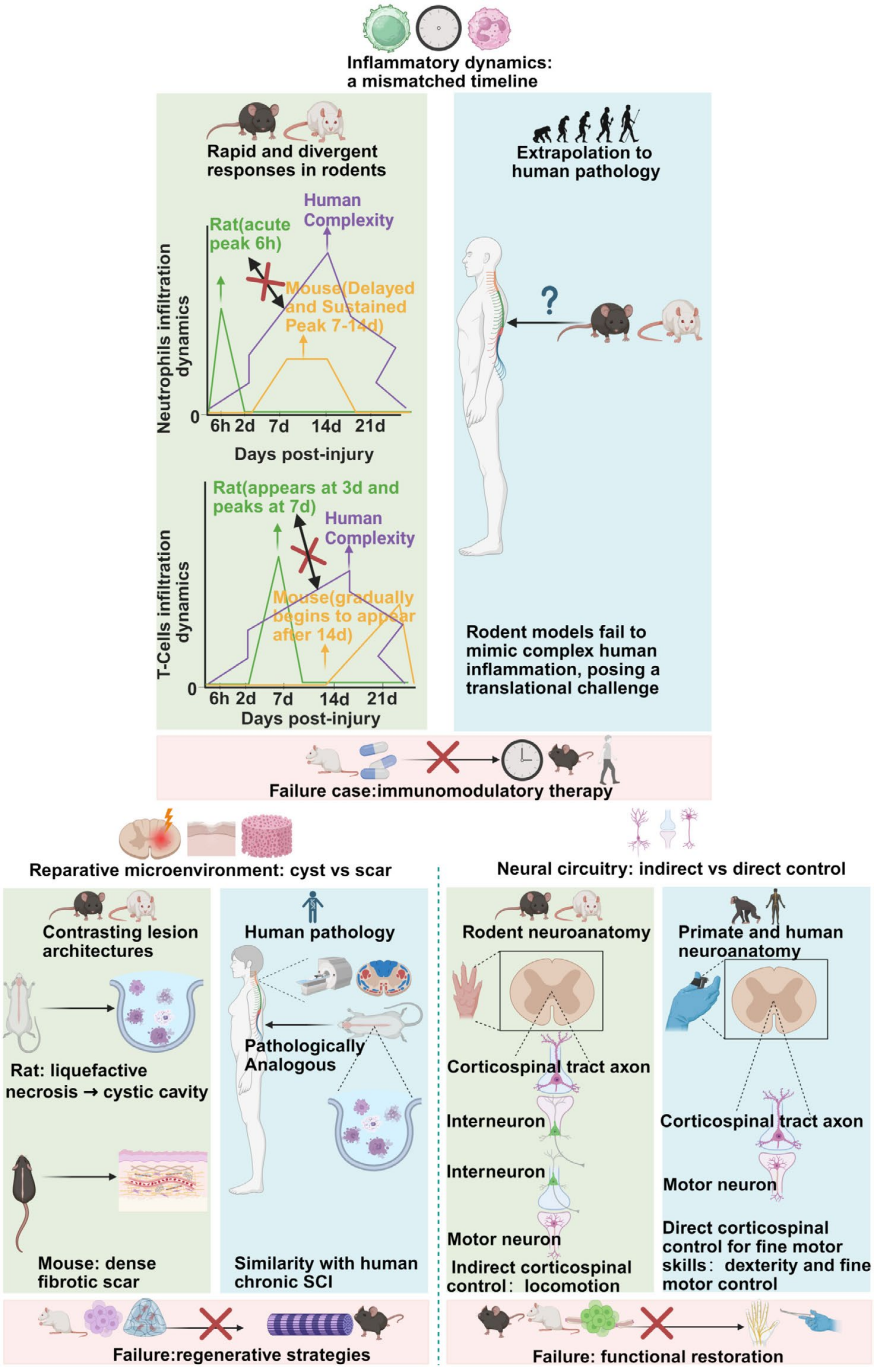
The three pillars of translational failure: interspecies gaps in immunodynamics, microenvironment, and neuroanatomy. This figure illustrates differences between rodent (rat/mouse) and human SCI across three key domains that impede clinical translation. Inflammatory dynamics mismatch: Significant discrepancies exist in the timeline of inflammatory cell (e.g., neutrophil and T cell) infiltration in rodents (e.g., neutrophil peak at 6 h in rats vs. 7–14 days in mice), which hinders the translation of immunomodulatory therapies. Repair microenvironment (cavity vs. scar): Rats tend to form cystic cavities, whereas mice develop dense fibrotic scars, the latter of which is pathologically more akin to human chronic SCI. This structural difference has led to translational failures for regenerative strategies. Neural circuitry (indirect vs. direct): Rodents primarily rely on indirect CST control, whereas humans and NHPs possess direct (monosynaptic) CST control that is critical for fine motor function. This anatomical difference impedes the translation of functional recovery strategies.

Although animal models of SCI successfully simulate the physical morphology of human trauma, the systemic failure of clinical translation reveals a more profound obstacle [95]. This barrier lies in the deep‐seated differences between species in the key biological responses that ultimately determine therapeutic success [13]. A substantial body of evidence indicates that these cross‐species pathophysiological chasms constitute the fundamental reason for translational research failure [14, 34, 35]. We will systematically dissect these differences from three core perspectives: inflammatory dynamics, the reparative microenvironment, and neuroanatomy (Figure 2).

### 3.1 The Temporal Mismatch in Inflammatory Dynamics

The temporal mismatch in postinjury inflammatory dynamics is a primary reason for the translational failure of immunomodulatory therapies. Rats and mice, the most commonly used SCI models, exhibit starkly different patterns of immune cell infiltration. In the rat contusion model, neutrophil infiltration peaks at 6 h postinjury and rapidly subsides within 48 h [35]. In mouse models, however, neutrophil numbers continue to increase, reaching their peak only between 3 and 14 days postinjury [34]. T‐cell infiltration shows a similarly significant delay: it occurs between 3 and 7 days in rats, whereas in mice, T‐cells are not detected until 14 days postinjury [14]. This fundamental difference in immunodynamics directly invalidates the therapeutic window across species. A drug designed to target acute‐phase (6‐h) neutrophil infiltration, proven effective in a rat model, is almost certainly doomed to fail if directly applied to a mouse model or extrapolated to humans, as it would miss the optimal window for intervention. Crucially, clinical neuropathological studies indicate that human SCI exhibits a distinct and more protracted inflammatory chronobiology. In human patients, neutrophil infiltration typically begins within hours but reaches its peak between 1 and 3 days postinjury, persisting for several weeks, while robust macrophage accumulation peaks at later stages (weeks to months) [96]. Consequently, neither the rapid, transient response in rats nor the significantly delayed onset in mice perfectly recapitulates the human condition; rather, the human inflammatory profile falls between these two rodent extremes while displaying a uniquely prolonged chronicity. This profound discrepancy dictates that relying exclusively on murine timelines to define clinical therapeutic windows is precarious. It further substantiates our argument that validation in large animal models (such as porcine models), which share closer immunological and temporal homology with humans, is an indispensable step before clinical translation.

### 3.2 Structural Differences in the Postinjury Reparative Microenvironment

Structural differences in the postinjury reparative microenvironment physically dictate the success or failure of regenerative medicine strategies. In the chronic phase, the lesion core in rats tends to liquefy, forming a cystic cavity that is pathologically similar to chronic human SCI [36, 97]. Conversely, the lesion core in mice predominantly becomes filled with a dense, fibrotic scar tissue rich in fibronectin and collagen, creating a physical barrier [13, 14]. While these phenotypes represent a biological spectrum rather than a strict dichotomy, the translational value lies in selecting the species that maximally recapitulates the specific pathological feature targeted by the therapy. Clinically, human SCI manifests as a heterogeneous, mixed phenotype comprising both cystic cavitation and dense fibrotic scarring. Quantitative neuropathological and advanced neuroimaging studies reveal that macroscopic cystic cavitation develops in approximately 50%–70% of severe human SCI cases, whereas dense fibrotic and glial scarring is virtually ubiquitous (nearly 100%) at the lesion epicenter or margins [98]. Therefore, rather than presenting “diametrically opposed” contradictions, these species‐specific divergences represent distinct facets of the complex human pathology. It is inaccurate to designate either species as the superior surrogate; instead, the rat model faithfully recapitulates the cystic component, while the mouse model mirrors the fibrotic barrier. This reality dictates that therapeutic evaluation must be mechanism‐specific: a strategy designed to fill a cystic void (rat‐appropriate) faces a fundamentally different physical challenge than one aiming to degrade a dense fibrotic matrix (mouse‐appropriate). Consequently, the selection of an animal model must be rigorously aligned with the specific pathological feature targeted by the intervention—cavity bridging versus scar modulation—rather than relying on a single species to represent the totality of human injury.

### 3.3 Innate Differences in Neural Circuit Anatomy

While the reparative microenvironment dictates the structural potential for axonal regrowth, the ultimate realization of function is governed by the wiring diagram itself. Here, innate differences in neuroanatomy fundamentally limit the translatability of rodent models in studies of functional recovery. The projection patterns of the CST, which is responsible for fine motor control, exhibit fundamental differences across species [59]. In rodents, the CST primarily projects to the dorsal horn of the spinal cord, indirectly modulating motor neurons [59]. In primates, including humans, however, the CST forms extensive monosynaptic connections that directly innervate motor neurons in the ventral horn. This anatomical feature is the basis for higher‐order functions such as fine, independent hand movements [59, 60]. This implies that the neural circuit reorganization underlying motor recovery observed in rodents may be entirely different from the mechanisms in humans. Consequently, while rodent models are invaluable for decoding propriospinal relay formation and plasticity‐mediated recovery, they possess an intrinsic validity ceiling for validating therapies specifically aiming to restore direct corticomotoneuronal control essential for human dexterity.

## 4 The Solution: Implementing the MFM Framework

**FIGURE 3:**
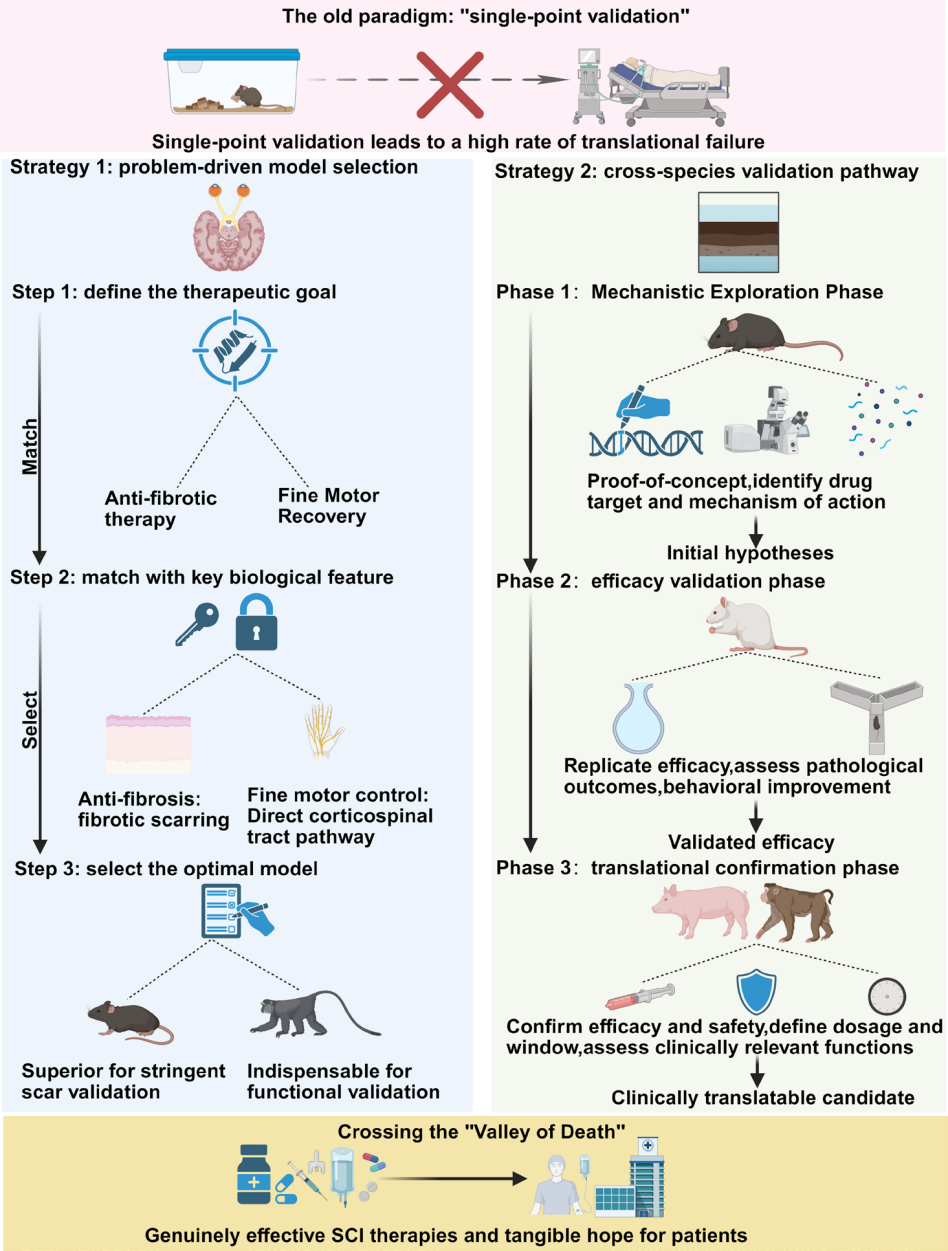
The mechanistic fidelity matching framework: integrating problem‐driven model selection with a hierarchical cross‐species validation pipeline. This schematic proposes a new framework to overcome the SCI translational “valley of death,” replacing the traditional “single‐point validation” (indicated by an “X” at the top). This framework comprises two core strategies. Question‐driven model selection (left panel): This emphasizes matching key biological features (e.g., fibrotic scarring) with specific therapeutic goals (e.g., anti‐fibrosis) to select the optimal model (e.g., mice for scar validation, primates for functional validation). Cross‐species validation pathway (right panel): This proposes a progressive three‐phase workflow: Phase 1 (mechanism exploration, e.g., mice), Phase 2 (efficacy validation, e.g., rats), Phase 3 (translational confirmation, e.g., NHPs), to systematically confirm efficacy, safety, and clinically relevant function. This integrated strategy aims to identify therapeutic candidates with clinical translation potential.

Given the profound chasm between the simulation of physical form and the reality of interspecies pathophysiological differences, the conventional one therapy, one model mindset of single‐point validation is no longer tenable. To systematically bridge this translational gap, we introduce the MFM framework. This paradigm posits that translational success depends not on finding a single universally valid surrogate, but on rigorously aligning the specific mechanism of action of a therapy with the biological feature of highest fidelity in a given model. Operationalizing MFM requires a departure from indiscriminate testing toward a structured matrix comprising two core strategies: problem‐driven model selection and a hierarchical cross‐species validation pathway (Figure 3).

### 4.1 Strategy 1: Problem‐Driven, Precise Model Selection

The cornerstone of this new paradigm is a shift from model‐driven to problem‐driven, precise model selection. The core mechanism of a given therapeutic strategy must be matched with the most relevant biological characteristic of the animal model. For instance, when developing antifibrotic and matrix‐remodeling strategies, the mouse model is superior. Because its lesion core is prone to forming a dense, fibrotic scar, it offers a more stringent and therefore more translationally valuable platform for validating therapies designed to inhibit scar formation [13]. Conversely, for therapies targeting functional recovery, particularly of fine motor control, ultimate validation in NHP models is indispensable. Only their CST anatomy and functional control patterns are sufficiently homologous to those of humans to provide effective predictive value for translation, especially for interventions aimed at restoring functions like hand dexterity [59, 60].

### 4.2 Strategy 2: A Multispecies, Multimodel Cross‐Validation Pathway

For a therapy to have a genuine potential for successful translation, its preclinical evidence chain must be reproducible across species, ensuring its efficacy is not contingent on the unique biological context of a single model. Here, we propose a strategic hierarchy, progressive cross‐validation pathway designed to systematically filter out false‐positive results. Crucially, this comprehensive validation need not be achieved by a single laboratory in isolation; rather, it should become a collective community standard for advancing candidate therapies to clinical trials. By leveraging multicenter collaborations and specialized core facilities, the field can distribute the burden of this rigorous pipeline. In the mechanistic exploration phase, the rich genetic tools available in mouse models should be leveraged to establish proof‐of‐concept at the molecular and cellular levels, definitively identifying the drug target and its mechanism of action. In the efficacy validation phase, the therapy should be advanced to rat models, where pathological features (such as cystic cavity formation) and behavioral assessment paradigms are more established. This stage serves as a crucial replication of the core therapeutic efficacy. In the translational confirmation phase (large animal validation), for therapies that demonstrate robust effects in both preceding stages, a final confirmation of efficacy, safety, dosage, and the therapeutic window is mandatory. This phase demands a differentiated approach: Porcine models provide a critical intermediate bridge, offering spinal cord dimensions and immune systems that approximate human physiology more closely than rodents while presenting a more economically and ethically accessible option for validating anti‐inflammatory agents or biomaterial scaffolds. However, for interventions specifically targeting the restoration of fine motor dexterity, NHPs remain the indispensable standard due to their unique direct corticospinal projections. Only by adhering to such a rigorous, multidimensional assessment paradigm can we screen for therapies that genuinely possess the potential to cross the valley of death, bringing tangible hope for an eventual cure for SCI.

## 5 Perspectives

**FIGURE 4:**
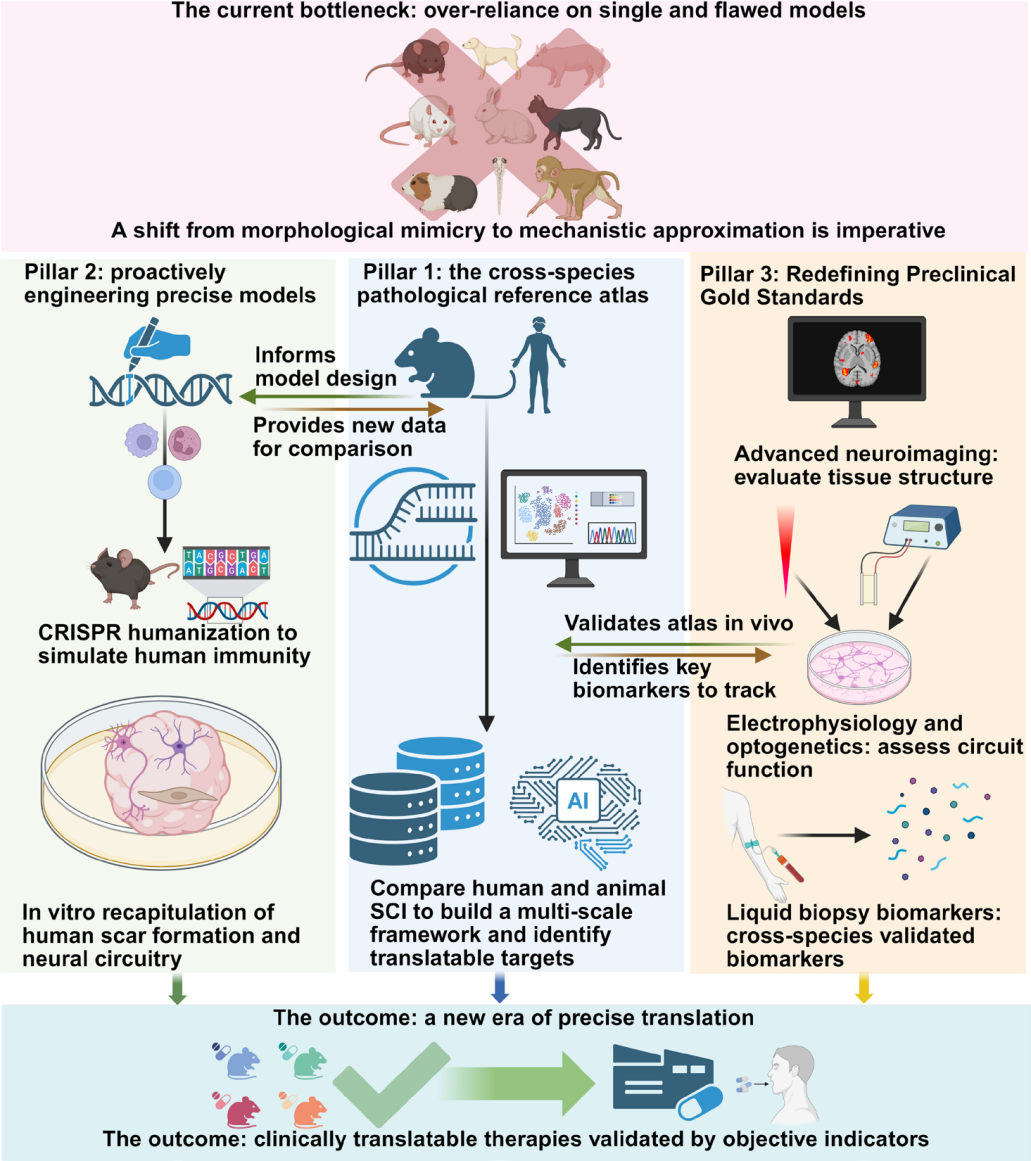
The future of SCI translation: integrating humanized models, multiomic atlases, and mechanism‐coupled biomarkers. The upper panel illustrates the current translational bottleneck resulting from an over‐reliance on single animal models that prioritize physical simulation over biological fidelity. To bridge this chasm, the schematic outlines a multidimensional research roadmap comprising three key initiatives. Pillar 1 (center): the construction of a cross‐species pathological reference atlas. This leverages high‐dimensional data (e.g., transcriptomics) and AI to systematically compare human and animal SCI, establishing a multiscale biological frame of reference to identify translatable targets. Pillar 2 (left): proactively engineering precise models. This shifts from passive selection to active design, utilizing CRISPR humanization to simulate human immune responses and in vitro organoids to recapitulate human‐specific scar formation and neural circuitry. Pillar 3 (right): redefining preclinical gold standards. This replaces subjective behavioral scoring with objective, mechanism‐coupled readouts, including advanced neuroimaging for tissue structure, electrophysiology for circuit function, and cross‐species validated liquid biopsy biomarkers. The bottom panel depicts the ultimate outcome: a new era of precise translation where therapies are validated by objective indicators to ensure clinical success.

To bridge the chasm between animal models and human pathology in preclinical SCI research, a paradigm shift from morphological mimicry to mechanistic approximation is imperative. The current reliance on single, flawed models has become a bottleneck for translational medicine; we must therefore pivot to a multidimensional research roadmap. The core of this new approach involves three key initiatives: constructing a cross‐species, quantitative pathological atlas to serve as a biological frame of reference; proactively engineering a new generation of more precise models; and revolutionizing preclinical assessment standards to prioritize mechanism‐based, objective biomarkers (Figure 4).

First, establishing a quantitative, cross‐species pathological reference atlas is the cornerstone of achieving precise translation. However, this effort must transcend the simple enumeration of cell types. We urgently need to leverage cutting‐edge technologies, such as spatial transcriptomics, to systematically dissect the spatial heterogeneity of the lesion microenvironment. A critical focus must be placed on the lesion border zone—the interface where regeneration succeeds or fails—to determine if the cellular neighborhoods and interactome networks in this region are evolutionarily conserved or divergent between rodents and humans. Furthermore, we propose a frontier shift toward the development of AI‐driven computational translation models (in silico modeling). By feeding these high‐dimensional cross‐species datasets into machine learning algorithms, we can move beyond qualitative comparison to algorithmic calibration. This would enable researchers to mathematically project how a therapeutic signal observed in the murine biological context would manifest in human physiology, effectively creating a computational bridge to predict clinical efficacy before trials begin.

Second, we must shift from passively selecting models to proactively engineering new models that more closely approximate human pathology. This requires the deep integration of gene‐editing and tissue‐engineering technologies. For example, using CRISPR to perform humanization modifications on key genes can create humanized mice that simulate human‐specific immune responses. Alternatively, assembloid in vitro models, built from organoids, can be used to more faithfully recapitulate the features of human scar formation and neural circuitry.

Finally, redefining the gold standard for preclinical research is critical to ensuring translational success. We must break our over‐reliance on behavioral scoring, as these outcomes are highly susceptible to interference from species‐specific compensatory mechanisms. Future efficacy validation must integrate a suite of objective, quantifiable assessment tools. These should include the use of advanced neuroimaging to evaluate tissue structure, electrophysiology, and optogenetics to assess circuit function, and the development of cross‐species‐validated liquid biopsy biomarkers. Only when a therapy demonstrates reproducible, positive effects across these mechanistic indicators can we have genuine confidence in its potential for clinical translation.

In conclusion, the value of preclinical inquiry lies not in isolating successful outcomes in murine models, but in forecasting clinical translatability. The implementation of the MFM framework allows us to deconstruct the fidelity paradox by matching specific therapeutic mechanisms to the species with the highest biological relevance. Consequently, evolutionary divergences are repurposed from confounding variables into a stratified validation matrix, thereby bridging the translational gap with genuine clinical candidates.

## Author Contributions

L.C. and M.X. conceived and designed the study. L.C., W.P., and Y.Z. analyzed the data and finalized the figures. L.C. wrote the manuscript. M.X. and V.Y. contributed to the paper re‐edit. All the authors revised and approved the final version of the manuscript.

## Funding

This work was supported by grants from Natural Science Foundation of Henan (262300421603); Henan Province Medical Science and Technology Research Program (SBGJ202403031); Postdoctoral Fellowship Program of China Postdoctoral Science Foundation (GZC20232401); Hunan Provincial Natural Science Foundation (2023JJ40572); National Natural Science Foundation of China (U25A2065, 82071331); Canadian Institutes of Health Research (VWY).

## Ethics Statement

The authors have nothing to report.

## Consent

The authors have nothing to report.

## Conflicts of Interest

The authors declare no conflicts of interest.

## Supporting information


**Figure S1:** Pathological mechanisms of SCI.
**Figure S2:** Preparation methods for spinal cord models in different animals.
**Figure S3:** Advantages and disadvantages of different SCI preparation methods.

## Data Availability

The data that support the findings of this study are available from the corresponding author upon reasonable request.

## References

[1] L. Cao , Z. Shangguan , Y. Zhang , et al., “Vegfr3 Activation of Pkd2l1+ CSF‐cNs Triggers the Neural Stem Cell Response in Spinal Cord Injury,” Cellular Signalling 130 (2025): 111675. 39986360 10.1016/j.cellsig.2025.111675

[2] Y. Ma , X. Yu , J. Pan , et al., “Exosomes: A Promising Microenvironment Modulator for Spinal Cord Injury Treatment,” International Journal of Biological Sciences 21 (2025): 3791–3824. 40520019 10.7150/ijbs.115242 PMC12160932

[3] N. Nagoshi , S. Hashimoto , H. Okano , and M. Nakamura , “Regenerative Medicine for Spinal Cord Injury Using Induced Pluripotent Stem Cells: From Animals to Humans,” Pain 165 (2024): S76–S81. 39560418 10.1097/j.pain.0000000000003306

[4] Z. Huang , J. Li , J. Wo , et al., “Intranasal Delivery of Brain‐Derived Neurotrophic Factor (BDNF)‐Loaded Small Extracellular Vesicles for Treating Acute Spinal Cord Injury in Rats and Monkeys,” Journal of Extracellular Vesicles 14 (2025): e70066. 40194993 10.1002/jev2.70066 PMC11975507

[5] S. Liu , Y. Dong , X. Zhang , et al., “Neuroprotection in Spinal Cord Ischemia‐Reperfusion Injury: Diosmetin's Role via TREM2‐Mediated Microglial Pyroptosis,” Free Radical Biology & Medicine 237 (2025): 369–382. 40482977 10.1016/j.freeradbiomed.2025.05.417

[6] K. Wang , X. Chen , M. Liu , et al., “Mitochondrial Dynamics Reveal Potential to Facilitate Axonal Regeneration After Spinal Cord Injury,” Journal of Translational Medicine 23 (2025): 617. 40457377 10.1186/s12967-025-06611-2 PMC12131810

[7] C. Zhang , S. Zhao , Z. Huang , et al., “Macropinocytosis Enhances Foamy Macrophage Formation and Cholesterol Crystallization to Activate NLRP3 Inflammasome After Spinal Cord Injury,” Redox Biology 79 (2024): 103469. 39700693 10.1016/j.redox.2024.103469 PMC11723182

[8] S. S. Chambel and C. D. Cruz , “Axonal Growth Inhibitors and Their Receptors in Spinal Cord Injury: From Biology to Clinical Translation,” Neural Regeneration Research 18 (2023): 2573–2581. 37449592 10.4103/1673-5374.373674 PMC10358698

[9] S. Kabu , Y. Gao , B. K. Kwon , and V. Labhasetwar , “Drug Delivery, Cell‐Based Therapies, and Tissue Engineering Approaches for Spinal Cord Injury,” Journal of Controlled Release: Official Journal of the Controlled Release Society 219 (2015): 141–154. 26343846 10.1016/j.jconrel.2015.08.060 PMC4656085

[10] K. Vajn , J. A. Plunkett , A. Tapanes‐Castillo , and M. Oudega , “Axonal Regeneration After Spinal Cord Injury in Zebrafish and Mammals: Differences, Similarities, Translation,” Neuroscience Bulletin 29 (2013): 402–410. 23893428 10.1007/s12264-013-1361-8 PMC5561943

[11] V. Dietz and M. E. Schwab , “From the Rodent Spinal Cord Injury Model to Human Application: Promises and Challenges,” Journal of Neurotrauma 34 (2016): 1826–1830. 27286800 10.1089/neu.2016.4513

[12] Y. S. Nout , E. S. Rosenzweig , J. H. Brock , et al., “Animal Models of Neurologic Disorders: A Nonhuman Primate Model of Spinal Cord Injury,” Neurotherapeutics: The Journal of the American Society for Experimental NeuroTherapeutics 9 (2012): 380–392. 22427157 10.1007/s13311-012-0114-0 PMC3337011

[13] A. Alizadeh , S. M. Dyck , and S. Karimi‐Abdolrezaee , “Traumatic Spinal Cord Injury: An Overview of Pathophysiology, Models and Acute Injury Mechanisms,” Frontiers in Neurology 10 (2019): 282. 30967837 10.3389/fneur.2019.00282 PMC6439316

[14] J. M. Sroga , T. B. Jones , K. A. Kigerl , V. M. McGaughy , and P. G. Popovich , “Rats and Mice Exhibit Distinct Inflammatory Reactions After Spinal Cord Injury,” Journal of Comparative Neurology 462 (2003): 223–240. 12794745 10.1002/cne.10736

[15] K. Fouad , P. G. Popovich , M. A. Kopp , and J. M. Schwab , “The Neuroanatomical‐Functional Paradox in Spinal Cord Injury,” Nature Reviews. Neurology 17 (2020): 53–62. 33311711 10.1038/s41582-020-00436-x PMC9012488

[16] J. J. Li , H. Liu , Y. Zhu , et al., “Animal Models for Treating Spinal Cord Injury Using Biomaterials‐Based Tissue Engineering Strategies,” Tissue Engineering. Part B, Reviews 28 (2021): 79–100. 33267667 10.1089/ten.TEB.2020.0267

[17] K. Verstappen , R. Aquarius , A. Klymov , et al., “Systematic Evaluation of Spinal Cord Injury Animal Models in the Field of Biomaterials,” Tissue Engineering, Part B, Reviews 28 (2022): 1169–1179. 34915758 10.1089/ten.teb.2021.0194 PMC9805871

[18] GBD Spinal Cord Injuries Collaborators , “Global, Regional, and National Burden of Spinal Cord Injury, 1990‐2019: A Systematic Analysis for the Global Burden of Disease Study 2019,” Lancet Neurology 22 (2023): 1026–1047. 37863591 10.1016/S1474-4422(23)00287-9 PMC10584692

[19] C. S. Ahuja , J. R. Wilson , S. Nori , et al., “Traumatic Spinal Cord Injury,” Nature Reviews. Disease Primers 3 (2017): 17018. 10.1038/nrdp.2017.18 28447605

[20] P. Freund , M. Seif , N. Weiskopf , et al., “MRI in Traumatic Spinal Cord Injury: From Clinical Assessment to Neuroimaging Biomarkers,” Lancet. Neurology 18 (2019): 1123–1135. 31405713 10.1016/S1474-4422(19)30138-3

[21] C. D. Gayen , M. A. Bessen , R. M. Dorrian , et al., “Survival Model of Thoracic Contusion Spinal Cord Injury in the Domestic Pig,” Journal of Neurotrauma 40 (2022): 965–980. 36200622 10.1089/neu.2022.0281

[22] R. U. Ahmed , D. Medina‐Aguinaga , S. Adams , et al., “Predictive Values of Spinal Cord Diffusion Magnetic Resonance Imaging to Characterize Outcomes After Contusion Injury,” Annals of Clinical Translational Neurology 10 (2023): 1647–1661. 37501362 10.1002/acn3.51855 PMC10502634

[23] J. Gao , M. K. Khang , Z. Liao , K. Webb , M. R. Detloff , and J. S. Lee , “Rolipram‐Loaded PgP Nanoparticle Reduces Secondary Injury and Enhances Motor Function Recovery in a Rat Moderate Contusion SCI Model,” Nanomedicine: Nanotechnology, Biology and Medicine 53 (2023): 102702. 37574117 10.1016/j.nano.2023.102702 PMC12207785

[24] C.‐B. Liu , D. G. Yang , J. Li , et al., “Diffusion Tensor Imaging Reveals Brain Structure Changes in Dogs After Spinal Cord Injury,” Neural Regeneration Research 18 (2023): 176–182. 35799539 10.4103/1673-5374.344839 PMC9241425

[25] X. Sun , L. Y. Huang , H. X. Pan , et al., “Bone Marrow Mesenchymal Stem Cells and Exercise Restore Motor Function Following Spinal Cord Injury by Activating PI3K/AKT/mTOR Pathway,” Neural Regeneration Research 18 (2023): 1067–1075. 36254995 10.4103/1673-5374.355762 PMC9827790

[26] J. A. Gruner , “A Monitored Contusion Model of Spinal Cord Injury in the Rat,” Journal of Neurotrauma 9 (1992): 123–128. 1404425 10.1089/neu.1992.9.123

[27] S.‐Y. Lee , B. D. Schmit , S. N. Kurpad , and M. D. Budde , “Acute Magnetic Resonance Imaging Predictors of Chronic Motor Function and Tissue Sparing in Rat Cervical Spinal Cord Injury,” Journal of Neurotrauma 39 (2022): 1727–1740. 35708112 10.1089/neu.2022.0034 PMC9734017

[28] Y. Li , Z. Lei , R. M. Ritzel , et al., “Impairment of Autophagy After Spinal Cord Injury Potentiates Neuroinflammation and Motor Function Deficit in Mice,” Theranostics 12 (2022): 5364–5388. 35910787 10.7150/thno.72713 PMC9330534

[29] Y. Li , R. M. Ritzel , N. Khan , et al., “Delayed Microglial Depletion After Spinal Cord Injury Reduces Chronic Inflammation and Neurodegeneration in the Brain and Improves Neurological Recovery in Male Mice,” Theranostics 10 (2020): 11376–11403. 33052221 10.7150/thno.49199 PMC7545988

[30] N. Wilkins , N. P. Skinner , A. Motovylyak , B. D. Schmit , S. Kurpad , and M. D. Budde , “Evolution of Magnetic Resonance Imaging as Predictors and Correlates of Functional Outcome After Spinal Cord Contusion Injury in the Rat,” Journal of Neurotrauma 37 (2020): 889–898. 31830856 10.1089/neu.2019.6731 PMC7071026

[31] Y. Xu , Y. Geng , H. Wang , et al., “Cyclic Helix B Peptide Alleviates Proinflammatory Cell Death and Improves Functional Recovery After Traumatic Spinal Cord Injury,” Redox Biology 64 (2023): 102767. 37290302 10.1016/j.redox.2023.102767 PMC10267601

[32] H. Zhang , Y. Chen , F. Li , et al., “Elamipretide Alleviates Pyroptosis in Traumatically Injured Spinal Cord by Inhibiting cPLA2‐Induced Lysosomal Membrane Permeabilization,” Journal of Neuroinflammation 20 (2023): 6. 36609266 10.1186/s12974-023-02690-4 PMC9825014

[33] A. Xu , Y. Yang , Y. Shao , M. Jiang , Y. Sun , and B. Feng , “FHL2 Regulates Microglia M1/M2 Polarization After Spinal Cord Injury via PARP14‐Depended STAT1/6 Pathway,” International Immunopharmacology 124 (2023): 110853. 37708708 10.1016/j.intimp.2023.110853

[34] K. A. Kigerl , V. M. McGaughy , and P. G. Popovich , “Comparative Analysis of Lesion Development and Intraspinal Inflammation in Four Strains of Mice Following Spinal Contusion Injury,” Journal of Comparative Neurology 494 (2006): 578–594. 16374800 10.1002/cne.20827 PMC2655318

[35] Y. Taoka , K. Okajima , M. Uchiba , et al., “Role of Neutrophils in Spinal Cord Injury in the Rat,” Neuroscience 79 (1997): 1177–1182. 9219976 10.1016/s0306-4522(97)00011-0

[36] Z. Li , S. Yu , X. Hu , et al., “Fibrotic Scar After Spinal Cord Injury: Crosstalk With Other Cells, Cellular Origin, Function, and Mechanism,” Frontiers in Cellular Neuroscience 15 (2021): 720938. 34539350 10.3389/fncel.2021.720938 PMC8441597

[37] H. Kumar , H. Choi , M. J. Jo , et al., “Neutrophil Elastase Inhibition Effectively Rescued Angiopoietin‐1 Decrease and Inhibits Glial Scar After Spinal Cord Injury,” Acta Neuropathologica Communications 6 (2018): 73. 30086801 10.1186/s40478-018-0576-3 PMC6080383

[38] H. Kumar , M. J. Jo , H. Choi , et al., “Matrix Metalloproteinase‐8 Inhibition Prevents Disruption of Blood‐Spinal Cord Barrier and Attenuates Inflammation in Rat Model of Spinal Cord Injury,” Molecular Neurobiology 55 (2017): 2577–2590. 28421532 10.1007/s12035-017-0509-3

[39] E. Giraldo , P. Bonilla , M. Mellado , et al., “Transplantation of Human‐Fetal‐Spinal‐Cord‐Derived NPCs Primed With a Polyglutamate‐Conjugated Rho/Rock Inhibitor in Acute Spinal Cord Injury,” Cells 11 (2022): 3304. 36291170 10.3390/cells11203304 PMC9600863

[40] J.‐R. Lee , J. W. Kyung , H. Kumar , et al., “Targeted Delivery of Mesenchymal Stem Cell‐Derived Nanovesicles for Spinal Cord Injury Treatment,” International Journal of Molecular Sciences 21 (2020): 4185. 32545361 10.3390/ijms21114185 PMC7312698

[41] V. Sarwahi , J. Galina , B. Thornhill , et al., “A Study of Critical Events That Lead to Spinal Cord Injury and the Importance of Rapid Reversal of Surgical Steps in Improving Neurological Outcomes: A Porcine Model,” Spine 45 (2020): E181–E188. 31513108 10.1097/BRS.0000000000003229

[42] S. van Gorp , M. Leerink , O. Kakinohana , et al., “Amelioration of Motor/Sensory Dysfunction and Spasticity in a Rat Model of Acute Lumbar Spinal Cord Injury by Human Neural Stem Cell Transplantation,” Stem Cell Research & Therapy 4 (2013): 57. 23710605 10.1186/scrt209 PMC3706882

[43] Y.‐K. Zhang , J. T. Liu , Z. W. Peng , et al., “Different TLR4 Expression and Microglia/Macrophage Activation Induced by Hemorrhage in the Rat Spinal Cord After Compressive Injury,” Journal of Neuroinflammation 10 (2013): 112. 24015844 10.1186/1742-2094-10-112 PMC3847110

[44] R. Navarro , S. Juhas , S. Keshavarzi , et al., “Chronic Spinal Compression Model in Minipigs: A Systematic Behavioral, Qualitative, and Quantitative Neuropathological Study,” Journal of Neurotrauma 29 (2012): 499–513. 22029501 10.1089/neu.2011.2076 PMC3278823

[45] P. D. Purdy , C. L. White 3rd , D. L. Baer , et al., “Percutaneous Translumbar Spinal Cord Compression Injury in Dogs From an Angioplasty Balloon: MR and Histopathologic Changes With Balloon Sizes and Compression Times,” AJNR. American Journal of Neuroradiology 25 (2004): 1435–1442. 15466348 PMC7975456

[46] J. Huang , X. Hu , Z. Chen , et al., “Fascin‐1 Limits Myosin Activity in Microglia to Control Mechanical Characterization of the Injured Spinal Cord,” Journal of Neuroinflammation 21 (2024): 88. 38600569 10.1186/s12974-024-03089-5 PMC11005239

[47] J. Lentilhas‐Graça , D. J. Santos , J. Afonso , et al., “The Secretome of Macrophages Has a Differential Impact on Spinal Cord Injury Recovery According to the Polarization Protocol,” Frontiers in Immunology 15 (2024): 1354479. 38444856 10.3389/fimmu.2024.1354479 PMC10912310

[48] Y. Luo , F. Yao , Y. Shi , et al., “Tocilizumab Promotes Repair of Spinal Cord Injury by Facilitating the Restoration of Tight Junctions Between Vascular Endothelial Cells,” Fluids and Barriers of the CNS 20 (2023): 1. 36624478 10.1186/s12987-022-00399-9 PMC9830903

[49] X. Quan , C. Yu , Z. Fan , et al., “Hydralazine Plays an Immunomodulation Role of Pro‐Regeneration in a Mouse Model of Spinal Cord Injury,” Experimental Neurology 363 (2023): 114367. 36858281 10.1016/j.expneurol.2023.114367

[50] A. N. Stewart , R. Kumari , W. M. Bailey , et al., “PTEN Knockout Using Retrogradely Transported AAVs Transiently Restores Locomotor Abilities in Both Acute and Chronic Spinal Cord Injury,” Experimental Neurology 368 (2023): 114502. 37558155 10.1016/j.expneurol.2023.114502 PMC10498341

[51] C. C. do Espírito Santo , F. Silva Fiorin , J. Ilha , M. M. M. F. Duarte , T. Duarte , and A. R. S. Santos , “Spinal Cord Injury by Clip‐Compression Induces Anxiety and Depression‐Like Behaviours in Female Rats: The Role of the Inflammatory Response,” Brain, Behavior, and Immunity 78 (2019): 91–104. 30659938 10.1016/j.bbi.2019.01.012

[52] Y.‐Q. Wu , J. Xiong , Z. L. He , et al., “Metformin Promotes Microglial Cells to Facilitate Myelin Debris Clearance and Accelerate Nerve Repairment After Spinal Cord Injury,” Acta Pharmacologica Sinica 43 (2021): 1360–1371. 34480113 10.1038/s41401-021-00759-5 PMC9160053

[53] S. P. Hui , A. Dutta , and S. Ghosh , “Cellular Response After Crush Injury in Adult Zebrafish Spinal Cord,” Developmental Dynamics: An Official Publication of the American Association of the Anatomists 239 (2010): 2962–2979. 10.1002/dvdy.22438 20931657

[54] X. Wang , Q. Gao , X. Yang , et al., “Long‐Term Anodal Block Stimulation at Sacral Anterior Roots Promoted Recovery of Neurogenic Bladder Function in a Rabbit Model of Complete Spinal Cord Injury,” Neural Regeneration Research 7 (2012): 352–358. 25774174 10.3969/j.issn.1673-5374.2012.05.005 PMC4350117

[55] M. Zurita , C. Aguayo , C. Bonilla , et al., “The Pig Model of Chronic Paraplegia: A Challenge for Experimental Studies in Spinal Cord Injury,” Progress in Neurobiology 97 (2012): 288–303. 22564435 10.1016/j.pneurobio.2012.04.005

[56] N. Cho , D. H. Nguyen , K. Satkunendrarajah , D. R. Branch , and M. G. Fehlings , “Evaluating the Role of IL‐11, a Novel Cytokine in the IL‐6 Family, in a Mouse Model of Spinal Cord Injury,” Journal of Neuroinflammation 9 (2012): 134. 22715999 10.1186/1742-2094-9-134 PMC3410772

[57] D. Han , Z. Yu , W. Liu , et al., “Plasma Hemopexin Ameliorates Murine Spinal Cord Injury by Switching Microglia From the M1 State to the M2 State,” Cell Death & Disease 9 (2018): 181. 29415995 10.1038/s41419-017-0236-8 PMC5833847

[58] Y. Gu , X. Cheng , X. Huang , et al., “Conditional Ablation of Reactive Astrocytes to Dissect Their Roles in Spinal Cord Injury and Repair,” Brain, Behavior, and Immunity 80 (2019): 394–405. 30959174 10.1016/j.bbi.2019.04.016

[59] E. S. Rosenzweig , G. Courtine , D. L. Jindrich , et al., “Extensive Spontaneous Plasticity of Corticospinal Projections After Primate Spinal Cord Injury,” Nature Neuroscience 13 (2010): 1505–1510. 21076427 10.1038/nn.2691 PMC3144760

[60] G. Courtine , M. B. Bunge , J. W. Fawcett , et al., “Can Experiments in Nonhuman Primates Expedite the Translation of Treatments for Spinal Cord Injury in Humans?,” Nature Medicine 13 (2007): 561–566. 10.1038/nm1595 PMC3245971 17479102

[61] A. H. All , S. Luo , X. Liu , and H. al‐Nashash , “Effect of Thoracic Spinal Cord Injury on Forelimb Somatosensory Evoked Potential,” Brain Research Bulletin 173 (2021): 22–27. 33991605 10.1016/j.brainresbull.2021.05.005

[62] Q. Han , Y. Xie , J. D. Ordaz , et al., “Restoring Cellular Energetics Promotes Axonal Regeneration and Functional Recovery After Spinal Cord Injury,” Cell Metabolism 31 (2020): 623–641.e8. 32130884 10.1016/j.cmet.2020.02.002 PMC7188478

[63] J. Nogueira‐Rodrigues , S. C. Leite , R. Pinto‐Costa , et al., “Rewired Glycosylation Activity Promotes Scarless Regeneration and Functional Recovery in Spiny Mice After Complete Spinal Cord Transection,” Developmental Cell 57 (2022): 440–450.e7. 34986324 10.1016/j.devcel.2021.12.008

[64] M. Zawadzka , M. Yeghiazaryan , S. Niedziółka , et al., “Forced Remyelination Promotes Axon Regeneration in a Rat Model of Spinal Cord Injury,” International Journal of Molecular Sciences 24 (2022): 495. 36613945 10.3390/ijms24010495 PMC9820536

[65] A. H. All and H. Al‐Nashash , “Comparative Analysis of Functional Assessment for Contusion and Transection Models of Spinal Cord Injury,” Spinal Cord 59 (2021): 1206–1209. 34493803 10.1038/s41393-021-00698-2

[66] A. R. Brown and M. Martinez , “Thoracic Spinal Cord Hemisection Surgery and Open‐Field Locomotor Assessment in the Rat,” Journal of Visualized Experiments: JoVE (2019). 10.3791/59738 31305520

[67] B.‐Q. Lai , R. J. Wu , W. T. Han , et al., “Tail Nerve Electrical Stimulation Promoted the Efficiency of Transplanted Spinal Cord‐Like Tissue as a Neuronal Relay to Repair the Motor Function of Rats With Transected Spinal Cord Injury,” Biomaterials 297 (2023): 122103. 37028111 10.1016/j.biomaterials.2023.122103

[68] Y. Li , M. Alam , S. Guo , K. H. Ting , and J. He , “Electronic Bypass of Spinal Lesions: Activation of Lower Motor Neurons Directly Driven by Cortical Neural Signals,” Journal of Neuroengineering and Rehabilitation 11 (2014): 107. 24990580 10.1186/1743-0003-11-107 PMC4094416

[69] A. Morales‐Guadarrama , H. Salgado‐Ceballos , I. Grijalva , et al., “Evolution of Spinal Cord Transection of Rhesus Monkey Implanted With Polymer Synthesized by Plasma Evaluated by Diffusion Tensor Imaging,” Polymers 14 (2022): 962. 35267785 10.3390/polym14050962 PMC8912689

[70] A. Paramasivam , S. Mickymaray , S. Jayakumar , et al., “Locomotor Behavior Analysis in Spinal Cord Injured *Macaca radiata* After Predegenerated Peripheral Nerve Grafting‐A Preliminary Evidence,” Veterinary Sciences 8 (2021): 288. 34941815 10.3390/vetsci8120288 PMC8707676

[71] S. Wilson , S. J. Nagel , L. A. Frizon , et al., “The Hemisection Approach in Large Animal Models of Spinal Cord Injury: Overview of Methods and Applications,” Journal of Investigative Surgery: The Official Journal of the Academy of Surgical Research 33 (2018): 240–251. 30380340 10.1080/08941939.2018.1492048

[72] X. Xue , X. Wu , Y. Fan , et al., “Heterogeneous Fibroblasts Contribute to Fibrotic Scar Formation After Spinal Cord Injury in Mice and Monkeys,” Nature Communications 15 (2024): 6321. 10.1038/s41467-024-50564-x PMC11282111 39060269

[73] D. M. Basso , L. C. Fisher , A. J. Anderson , L. B. Jakeman , D. M. Mctigue , and P. G. Popovich , “Basso Mouse Scale for Locomotion Detects Differences in Recovery After Spinal Cord Injury in Five Common Mouse Strains,” Journal of Neurotrauma 23 (2006): 635–659. 16689667 10.1089/neu.2006.23.635

[74] B. Burris , N. Jensen , and M. H. Mokalled , “Assessment of Swim Endurance and Swim Behavior in Adult Zebrafish,” Journal of Visualized Experiments: JoVE (2021). 10.3791/63240 PMC9226856 34842242

[75] Z. Ji , Z.‐L. Zhou , Q. Hao , et al., “Activating Transcription Factor 6 Contributes to Functional Recovery After Spinal Cord Injury in Adult Zebrafish,” Journal of Molecular Neuroscience: MN 71 (2020): 734–745. 32895880 10.1007/s12031-020-01691-9

[76] A. Kimura , K. Suehiro , A. Mukai , et al., “Protective Effects of Hydrogen Gas Against Spinal Cord Ischemia‐Reperfusion Injury,” Journal of Thoracic and Cardiovascular Surgery 164 (2021): e269–e283. 34090694 10.1016/j.jtcvs.2021.04.077

[77] S. Lv , K. Zhao , R. Li , C. Meng , G. Li , and F. Yin , “EGFR‐Activated JAK2/STAT3 Pathway Confers Neuroprotection in Spinal Cord Ischemia‐Reperfusion Injury: Evidence From High‐Throughput Sequencing and Experimental Models,” Molecular Neurobiology 61 (2023): 646–661. 37656314 10.1007/s12035-023-03548-9

[78] K. Yamanaka , M. Eldeiry , M. Aftab , et al., “Pretreatment With Diazoxide Attenuates Spinal Cord Ischemia‐Reperfusion Injury Through Signaling Transducer and Activator of Transcription 3 Pathway,” Annals of Thoracic Surgery 107 (2018): 733–739. 30395862 10.1016/j.athoracsur.2018.09.031

[79] N. Jing , B. Fang , Z. Li , and A. Tian , “Exogenous Activation of Cannabinoid‐2 Receptor Modulates TLR4/MMP9 Expression in a Spinal Cord Ischemia Reperfusion Rat Model,” Journal of Neuroinflammation 17 (2020): 101. 32248810 10.1186/s12974-020-01784-7 PMC7132899

[80] Z. Xu and Z. Li , “Experimental Study on the Role of Apelin‐13 in Alleviating Spinal Cord Ischemia Reperfusion Injury Through Suppressing Autophagy,” Drug Design, Development and Therapy 14 (2020): 1571–1581. 32368015 10.2147/DDDT.S241066 PMC7183780

[81] W. Yang , Q. Q. Wu , L. Yang , et al., “Awake Rabbit Model of Ischemic Spinal Cord Injury With Delayed Paraplegia: The Role of Ambient Temperature,” Animal Models and Experimental Medicine 7 (2023): 732–739. 37697489 10.1002/ame2.12346 PMC11528386

[82] C. R. Behem , T. Friedheim , S. H. Wipper , et al., “Real‐Time Assessment of Spinal Cord Microperfusion in a Porcine Model of Ischemia/Reperfusion,” Journal of Visualized Experiments: JoVE (2020). 10.3791/62047 33369603

[83] K. Kanda , O. Adachi , S. Kawatsu , et al., “Oxygenation of the Cerebrospinal Fluid With Artificial Cerebrospinal Fluid Can Ameliorate a Spinal Cord Ischemic Injury in a Rabbit Model,” Journal of Thoracic and Cardiovascular Surgery 152 (2016): 1401–1409. 27640950 10.1016/j.jtcvs.2016.04.095

[84] H. Li , X. Dong , Y. Yang , M. Jin , and W. Cheng , “The Neuroprotective Mechanism of Spinal Cord Stimulation in Spinal Cord Ischemia/Reperfusion Injury,” Neuromodulation: Journal of the International Neuromodulation Society 24 (2020): 43–48. 32114698 10.1111/ner.13113

[85] D. Mazensky , S. Flesarova , and I. Sulla , “Arterial Blood Supply to the Spinal Cord in Animal Models of Spinal Cord Injury. A Review,” Anatomical Record 300 (2017): 2091–2106. 10.1002/ar.23694 28972696

[86] D. Y. Yoo , S. B. Cho , H. Y. Jung , et al., “Tat‐Protein Disulfide‐Isomerase A3: A Possible Candidate for Preventing Ischemic Damage in the Spinal Cord,” Cell Death & Disease 8 (2017): e3075. 28981094 10.1038/cddis.2017.473 PMC5680594

[87] Q. J. Yu and Y. Yang , “Function of SOD1, SOD2, and PI3K/AKT Signaling Pathways in the Protection of Propofol on Spinal Cord Ischemic Reperfusion Injury in a Rabbit Model,” Life Sciences 148 (2016): 86–92. 26851534 10.1016/j.lfs.2016.02.005

[88] H. Li , P. Wang , L. Tang , et al., “Distinct Polarization Dynamics of Microglia and Infiltrating Macrophages: A Novel Mechanism of Spinal Cord Ischemia/Reperfusion Injury,” Journal of Inflammation Research 14 (2021): 5227–5239. 34675600 10.2147/JIR.S335382 PMC8521441

[89] Z. Ma , Q. Dong , B. Lyu , J. Wang , Y. Quan , and S. Gong , “The Expression of Bradykinin and Its Receptors in Spinal Cord Ischemia‐Reperfusion Injury Rat Model,” Life Sciences 218 (2018): 340–345. 30580020 10.1016/j.lfs.2018.12.034

[90] N. Ning , X. Dang , C. Bai , C. Zhang , and K. Wang , “Panax Notoginsenoside Produces Neuroprotective Effects in Rat Model of Acute Spinal Cord Ischemia‐Reperfusion Injury,” Journal of Ethnopharmacology 139 (2011): 504–512. 22154967 10.1016/j.jep.2011.11.040

[91] M. Tokmak , Y. Yuksel , M. H. Sehitoglu , et al., “The Neuroprotective Effect of Syringic Acid on Spinal Cord Ischemia/Reperfusion Injury in Rats,” Inflammation 38 (2015): 1969–1978. 25903968 10.1007/s10753-015-0177-2

[92] P. D. Smith , F. Puskas , X. Meng , et al., “The Evolution of Chemokine Release Supports a Bimodal Mechanism of Spinal Cord Ischemia and Reperfusion Injury,” Circulation 126 (2012): S110–S117. 22965970 10.1161/CIRCULATIONAHA.111.080275

[93] A. Jiao , C. Zhang , X. Wang , et al., “Single‐Cell Sequencing Reveals the Evolution of Immune Molecules Across Multiple Vertebrate Species,” Journal of Advanced Research 55 (2023): 73–87. 36871615 10.1016/j.jare.2023.02.017 PMC10770119

[94] G. Sollberger , A. J. Brenes , J. Warner , J. S. C. Arthur , and A. J. M. Howden , “Quantitative Proteomics Reveals Tissue‐Specific, Infection‐Induced and Species‐Specific Neutrophil Protein Signatures,” Scientific Reports 14 (2024): 5966. 38472281 10.1038/s41598-024-56163-6 PMC10933280

[95] M. T. Bell , F. Puskas , V. A. Agoston , et al., “Toll‐Like Receptor 4‐Dependent Microglial Activation Mediates Spinal Cord Ischemia‐Reperfusion Injury,” Circulation 128 (2013): S152–S156. 24030400 10.1161/CIRCULATIONAHA.112.000024

[96] T. Zrzavy , C. Schwaiger , I. Wimmer , et al., “Acute and Non‐Resolving Inflammation Associate With Oxidative Injury After Human Spinal Cord Injury,” Brain 144 (2021): 144–161. 33578421 10.1093/brain/awaa360 PMC7880675

[97] G. A. Metz , G. Metz , A. Curt , et al., “Validation of the Weight‐Drop Contusion Model in Rats: A Comparative Study of Human Spinal Cord Injury,” Journal of Neurotrauma 17 (2000): 1–17. 10674754 10.1089/neu.2000.17.1

[98] B. A. Kakulas , “The Applied Neuropathology of Human Spinal Cord Injury,” Spinal Cord 37 (1999): 79–88. 10065745 10.1038/sj.sc.3100807

