# Supplementary material for: The Fidelity Paradox in Spinal Cord Injury: Reframing Biomechanical Mimicry and Neurobiological Relevance for Clinical Translation
Source: CNS Neurosci Ther. 2026 May 16;32(5):e70929. doi: 10.1002/cns.70929 (PMC13179581; doi:10.1002/cns.70929)

**Supplementary figures**

**Supplementary figure 1. Pathological mechanisms of SCI.**

This figure illustrates the progression following SCI from the acute phase (0–48h) to the subacute phase (2d–2w) and to the chronic phase (>2w). The acute phase is characterized by blood-spinal cord barrier (BSCB) disruption, inflammatory infiltration (neutrophils and monocytes), microglial activation (releasing TNF-α and IL-1β), and excitotoxicity (glutamate, ROS, Ca2+). The subacute phase involves T cell infiltration, myelin debris clearance (phagocytosis by microglia and macrophages), and astrocyte activation. The chronic phase, in turn, is marked by reactive astrocytes and fibroblasts jointly forming an inhibitory glial-fibrotic scar.

**Supplementary figure 2. Preparation methods for spinal cord models in different animals.**

(a) Compressive SCl model: the compression method for mice apply a regtangular plate and modified SPI cortex tension/compression gauge; the compression method for rat apply a mental rod and catheter with a balloon fill with saline; the compression method for cat apply a weight of 180 grams and diameter screw; the compression method for dog apply a balloon dilation catheter and precalibrated hydraulic piston loading device; the compression method for pig apply a circular rod and balloon with saline. (b) Transverse SCI model: the cross-sectional methods for mice, rats, guinea pigs, rabbits, cats, dogs, pigs, primates, and zebrafish are that tools such as scissors, surgical knives, suction devices, or fine needles are used to achieve either hemisection or complete transection of the spinal cord. (c) Contusion SCI model: the impact methods for mice, rats, guinea pigs, rabbits, dogs, pigs, and primates are that contusion modeling of different levels of SCI using different weights of impactors free-falling from different heights or using different impactor. (d) Crush SCl model: The preparation methods for mice, rats, guinea pigs, rabbits, pigs, and zebrafish are that different kinds of clamps are used to achieve crush of the spinal cord. (e) Ischemic SCl model: The preparation methods for mice, rats, guinea pigs, rabbits, dogs, pigs, and primates are that ischemic SCI induced by clamping the decreasing aorta with an arterial clip or inserting the left femoral artery with a fogarty balloon catheter.

**Supplementary figure 3. Advantages and disadvantages of different SCI preparation methods.**

This schematic compares the five major SCI models: compressive, contusion, crush, ischemic, and transverse. The diagram employs a radial layout, with five uniquely colored branches representing each model. Each branch, moving from the center outward, illustrates the modeling method, key advantages (identified by a green checkmark), and major limitations (indicated by a red warning icon).

**Supplementary figure 1**


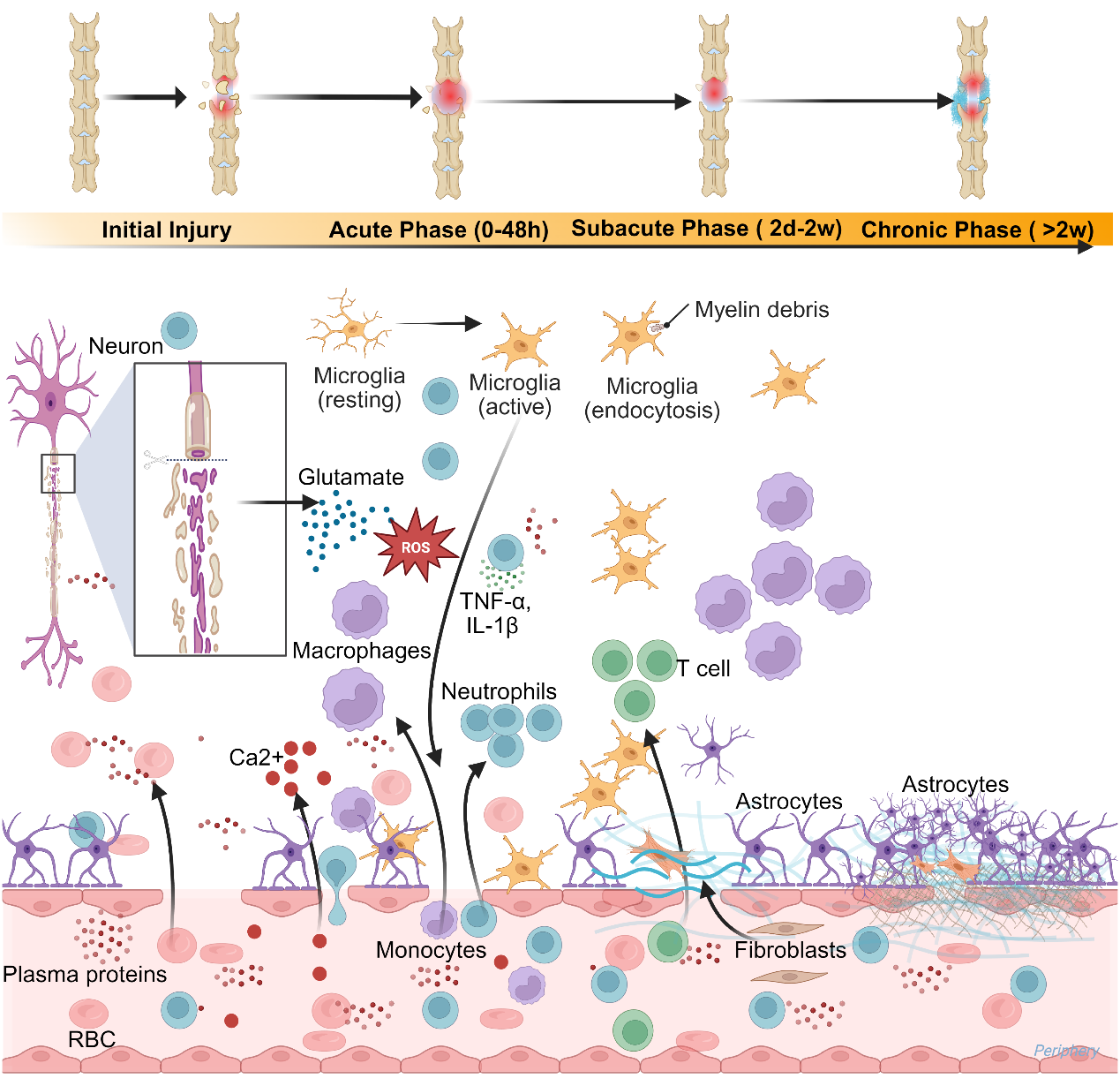


**Supplementary figure 2**

**
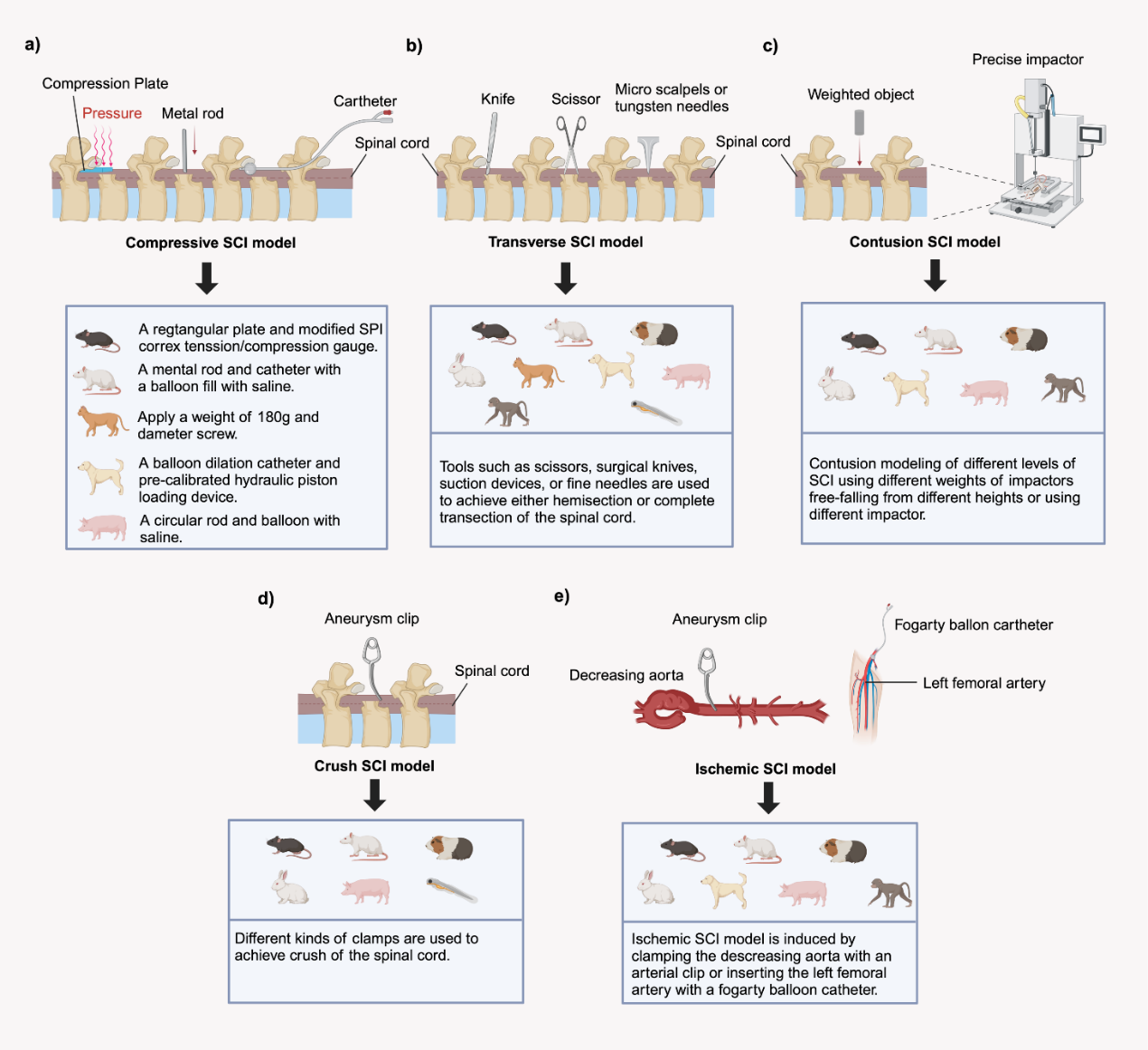
**

**Supplementary figure 3**


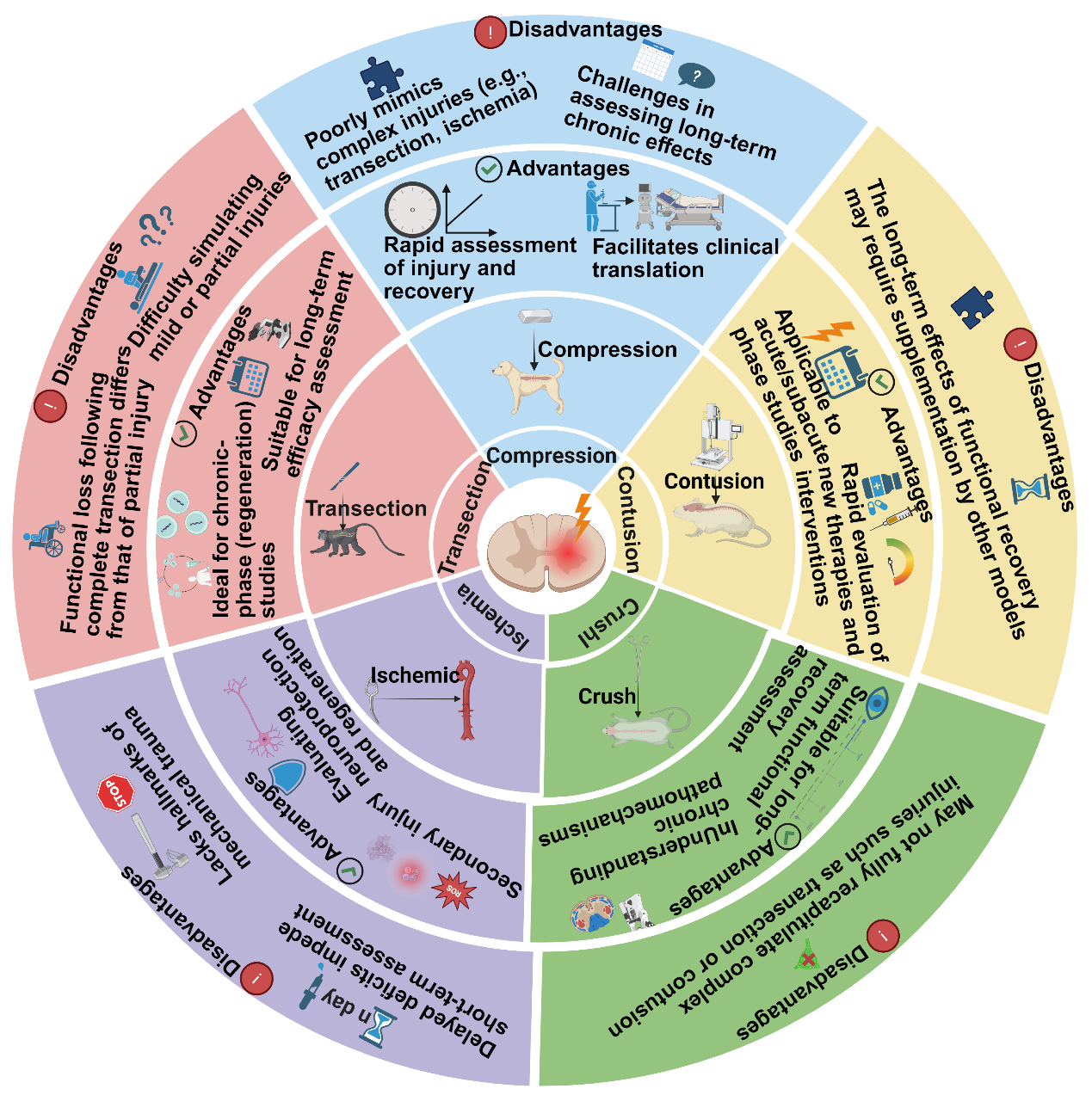

Supplement: Supplementary file 1 — Figure S1: Pathological mechanisms of SCI. Figure S2: Preparation methods for spinal cord models in different animals. Figure S3: Advantages and disadvantages of different SCI preparation methods. [file CNS-32-e70929-s001.docx]
